# Alteration of the anatomical covariance network after corpus callosotomy in pediatric intractable epilepsy

**DOI:** 10.1371/journal.pone.0222876

**Published:** 2019-12-05

**Authors:** Riyo Ueda, Hiroshi Matsuda, Noriko Sato, Masaki Iwasaki, Daichi Sone, Eri Takeshita, Yuko Shimizu-Motohashi, Akihiko Ishiyama, Takashi Saito, Hirofumi Komaki, Eiji Nakagawa, Kenji Sugai, Masayuki Sasaki, Yoshimi Kaga, Hiroshige Takeichi, Masumi Inagaki

**Affiliations:** 1 Department of Developmental Disorders, National Institute of Mental Health, National Center of Neurology and Psychiatry, Tokyo, Japan; 2 Division of Frontier Medicine and Pharmacy, Graduate School of Medical and Pharmaceutical Science, Chiba University, Chiba, Japan; 3 Integrative Brain Imaging Center, National Center of Neurology and Psychiatry, Tokyo, Japan; 4 Department of Radiology, National Center Hospital, National Center of Neurology and Psychiatry, Tokyo, Japan; 5 Department of Neurosurgery, National Center Hospital, National Center of Neurology and Psychiatry, Tokyo, Japan; 6 Department of Clinical and Experimental Epilepsy, UCL Institute of Neurology, London, England, United Kingdom; 7 Department of Child Neurology, National Center Hospital, National Center of Neurology and Psychiatry, Tokyo, Japan; 8 Computational Engineering Applications Unit, RIKEN, Wako, Japan; University of Modena and Reggio Emilia, ITALY

## Abstract

**Purpose:**

This study aimed to use graph theoretical analysis of anatomical covariance derived from structural MRI to reveal how the gray matter connectivity pattern is altered after corpus callosotomy (CC).

**Materials and methods:**

We recruited 21 patients with epilepsy who had undergone CC. Enrollment criteria were applied: (1) no lesion identified on brain MRI; (2) no history of other brain surgery; and (3) age not younger than 3 years and not older than 18 years at preoperative MRI evaluation. The most common epilepsy syndrome was Lennox-Gastaut syndrome (11 patients). For voxel-based morphometry, the normalized gray matter images of pre-CC and post-CC patients were analyzed with SPM12 (voxel-level threshold of p<0.05 [familywise error-corrected]). Secondly, the images of both groups were subjected to graph theoretical analysis using the Graph Analysis Toolbox with SPM8. Each group was also compared with 32 age- and sex-matched control patients without brain diseases.

**Results:**

Comparisons between the pre- and post-CC groups revealed a significant reduction in seizure frequency with no change in mean intelligence quotient/developmental quotient levels. There was no relationship among the three groups in global network metrics or in targeted attack. A regional comparison of betweenness centrality revealed decreased connectivity to and from the right middle cingulate gyri and medial side of the right superior frontal gyrus and a partial shift in the distribution of betweenness centrality hubs to the normal location. Significantly lower resilience to random failure was found after versus before CC and versus controls (p = 0.0450 and p = 0.0200, respectively).

**Conclusion:**

Graph theoretical analysis of anatomical covariance derived from structural imaging revealed two neural network effects of resection associated with seizure reduction: the reappearance of a structural network comparable to that in healthy children and reduced connectivity along the median line, including the middle cingulate gyrus.

## Introduction

Corpus callosotomy (CC) is an established palliative treatment for patients with intractable epilepsy who are not candidates for resective surgery. In a prospective population-based observational study, a greater than 50% seizure reduction was seen in 64% of the patients in the long-term follow-up, including 10% with seizure freedom [1]. The most common indication for CC is drop attacks [2].

Total CC patients show a higher reduction in seizures than patients who undergo partial CC [2]. Three factors related to seizure freedom after CC are “lack of abnormal magnetic resonance imaging findings”, “lack of proven etiology of seizures”, and underwent “surgery at age 6 years or younger” [3]. Regarding developmental outcomes, the sequential mean developmental quotient (DQ) score during a 3-year follow-up after CC was not significantly changed in children who underwent CC or vagal nerve stimulation [4]. Patients even show attention enhancement, which is related to drop seizure frequency and postoperative EEG improvements [5, 6].

It is largely unclear, however, why CC is effective for seizure and maladaptive behavior improvement and what kind of neural basis it alters. On the other hand, there were some effects of surgery in retrospective observational studies [1, 5, 6]. Brain functioning is increasingly seen as a complex interplay of dynamic neural systems that rely on the integrity of structural and functional networks [7]. In this context, epilepsy has been considered to arise from a dysfunction of neural networks and there have been investigations of the neural network alterations after CC using a 19-channel electroencephalography acquisition system in patients with Lennox-Gastaut syndrome (LGS) [8, 9]. These studies found that the hubs moved from the paramedian regions to the dual-hemisphere and even the lateral regions and that small-worldness increased in patients after successful CC. The covariation of small-worldness with the rate of the reduction in seizure frequency suggested that it can be used as an indicator of CC outcome.

Graph theoretical analysis is a branch of mathematics whose central idea is to represent a complex set of relationships with a set of nodes and their connections [10]. In the modern network science of neurological disorders (including epilepsy), functional magnetic resonance imaging (MRI) can be used to assess the time course of blood oxygen level-dependent signals and diffusion tensor imaging (DTI) can estimate whole-brain white matter networks using fiber tractography [7]. Anatomical covariance is another useful analysis methodology that uses standard structural MRI [11]. In a set of brain regions, either cortical thickness or cortical gray matter volumes can be quantified on three-dimensional (3D) T1-weighted MRI [12]. Briefly, gray matter volume changes in one brain region are significantly correlated with changes in other brain regions. Indeed, graph theoretical analysis has been applied to structural MRI for the evaluation of networks using regional gray matter volumes, which represents a new opportunity for understanding the brain as a complex system of interacting units and epilepsy as a modification of the system [7, 10]. Both focal epilepsy and generalized epilepsy patients exhibit marked alterations in neural networks versus healthy adults or children, and shifts in network topology have been associated with clinically relevant parameters, including disease duration and cognitive function, suggesting a possible utility of topological markers in diagnosis and management [13–16]. Thus, graph theoretical analysis using structural MRI has the considerable potential to reveal the neural network improvements induced by CC.

The present study aimed to reveal alterations in the structure of the neural network after CC. Graph theoretical analysis using structural MRI is a reasonable approach for a number of reasons. The first is that the corpus callosum is the largest white matter structure in the brain and the major commissural pathway connecting cortical regions of both hemispheres [17]. The second reason is that the above-mentioned clinical improvement after CC suggested the possibility of neural network alterations [1, 5]. The third reason is that most CC candidates with severe intellectual disability undergo structural MRI as part of the clinical examination.

In this study, we applied graph theoretical analysis to the whole gray matter network. We clarified the postoperative network alterations in pediatric intractable epilepsy patients and compared the results with those of previous studies.

## Materials and methods

### Participants

From a total of 86 patients, 21 were selected in accordance with the following inclusion criterion: patients who underwent CC between 2010 and 2017 at the National Center Hospital, National Center for Neurology and Psychiatry (NCNP). The following enrollment criteria were applied: (1) no lesion identified on brain MRI; (2) no history of other brain surgery; and (3) age not younger than 3 years and not older than 18 years at preoperative MRI evaluation (S1 Fig). The classifications of epilepsy syndromes were 11 patients with LGS, 7 patients with early-onset epileptic encephalopathy, 1 patient with epilepsy with myoclonic seizures, and 2 patients with unclassified syndrome. The classifications of epilepsy etiologies were 3 patients with gene abnormalities, 1 patient with infectious etiology, and 17 patients with unknown etiologies. Mean age at seizure onset was 2.3 (median 1.3, 0.2–12.6) years, and mean age at surgery was 8.6 (standard deviation [SD] 4.3) years. Preoperative investigation involving video electroencephalogram monitoring, MRI, PET-CT, and magnetoencephalography confirmed intractable epilepsy without structural abnormalities in all patients. Their electroencephalograms showed bilateral epileptiform discharges such as diffuse slow spike-and-wave or multi-focal spikes. The clinical data from the medical chart used in this study included sex, age, age at seizure onset, seizure frequency, antiepileptic drugs, sequential intelligence quotient (IQ) or DQ levels on the Wechsler Intelligence Scale for Children Third Edition (WISC-III) and Fourth Edition (WISC-IV) or the Kinder Infant Development Scale (KIDS), and period of MRI evaluation [18].

In addition, 32 age-matched patients with no central nervous system abnormality were recruited to comprise the control group in a conventional manner [19, 20]. The most common diagnosis in control patients was orthostatic hypotension (10 patients). There were 3 patients with somatoform disorder, 3 with congenital hypotonia, 3 with febrile seizure, 2 with day dreaming, 2 with motion sickness, 2 with nonpathologic lower limb hypertonia, 1 with masturbation, 1 with head circumference enlargement, and 1 with hereditary peripheral neuropathy. All control patients were confirmed to be normal on neurological examination by a board-certified child neurologist. Patients with a final diagnosis of somatoform disorders, congenital hypotonia, nonpathologic lower limb hypertonia, nonpathologic transient hallucination, or amnesia were monitored to track the improvements in their symptoms.

This retrospective study was approved by the Institutional Review Board at the National Center of Neurology and Psychiatry (A2017-060), and the need for informed consent was waived.

### MRI acquisitions, processing, and morphometry

The framework of the analysis is the same as in Takeda et al. [19] and Sone et al. [21]. Brain MRI was acquired twice in the patients—preoperatively (pre-CC data group) and postoperatively (mean 4.5 months after CC, post-CC data group)—and once in the control group using a 3.0-T MR system with a 32-channel coil (Philips Medical Systems, Best, The Netherlands). Three-dimensional sagittal T1-weighted magnetization-prepared rapid acquisition with gradient echo (MPRAGE) images were acquired with the following parameters: repetition time (TR)/echo time (TE), 7.12 ms/3.4 ms; flip angle, 10°; number of excitations (NEX), 1; 0.6-mm effective slice thickness with no gap; slices, 300; matrix, 260 × 320; and field of view (FOV), 26 × 24 cm. Additionally, transverse conventional T1-weighted images, transverse turbo spin echo T2-weighted images, and coronal fluid-attenuated inversion recovery images were acquired to determine the possible presence of any brain structural abnormalities.

Subsequently, standard voxel-based morphometry was performed on the 3D T1-weighted MPRAGE images. Namely, the images were segmented into gray matter, white matter, and cerebrospinal fluid by a unified tissue segmentation procedure after image intensity nonuniformity correction. The segmented gray and white matter images were spatially normalized to a customized template in standardized anatomic space using Diffeomorphic Anatomical Registration through Exponentiated Lie (DARTEL) [22]. Each image was modulated by the Jacobian determinant derived from the spatial normalization by DARTEL and spatially smoothed with an 8-mm full-width at half-maximum Gaussian kernel to decrease spatial noise and compensate for imprecise normalization [19, 21]. The morphological differences between the pre-CC and post-CC patient data groups and between the pre-CC and control groups were confirmed by submitting the resulting gray matter images after normalization using the Computational Anatomy Toolbox (CAT12) (http://www.neuro.uni-jena.de/cat/), which runs within SPM12. The morphological differences between the pre-CC and post-CC patient data groups were evaluated using the improved algorithms with CAT12 for longitudinal voxel-based morphometry analyses. The morphological differences between the pre-CC and control groups were also evaluated using CAT12 for cross-sectional analyses. These comparisons were performed with paired *t*-test analyses in SPM12. Differences meeting a voxel-level threshold of p<0.05 (FWE-corrected) and a cluster size criterion > 50 voxels were deemed significant.

### Graph theoretical analysis

The normalized gray matter images of each data group were submitted to graph theoretical analysis. For each group, the structural correlation network was constructed by analyzing a total of 90 cortical and subcortical regions of interest (ROIs) in the Automated Anatomical Labeling template [23] to form a 90 × 90 association matrix consisting of Pearson correlation coefficients between cortical gray matter volumes (i.e., anatomical covariance). Then, threshold maps were converted to binary adjacency maps with values of 0 or 1, with thresholds set across a range of network densities (*D*_min_–*D*_max_), where *D*_min_ (in this study, *D*_min_ = 0.1 and *D*_max_ = 0.5 at intervals of 0.02) means the lowest density that can permit a fully connected network [22]. Network hubs were identified and quantified based on measures of betweenness centrality (BC) and degree distribution. While degree distribution represents the abundance of connections to and from a region (the number of edges for a node), BC represents the abundance of more direct connections to and from a region (the number of shortest paths for a node, see van Diessen et al. [7]). Additionally, the following network metrics were calculated: clustering coefficient (*C*), a measure of the number of edges between nearest neighbors of nodes; characteristic path length (*L*), the average shortest path length between all pairs of nodes as a measure of network integration; global efficiency (*E*_*glob*_), the exchange of information across the whole network, which is inversely related to the path length; local efficiency (*E*_*loc*_), the inverse of the average shortest path connecting neighbors of nodes; small-worldness (*σ*), an indicator of ensemble topology, which is calculated as [*C*/C_rand_]/[*L*/L_rand_], where C_rand_ and L_rand_, are the mean clustering coefficient and the characteristic path length of 20 random networks, respectively; and modularity, a measure of the strength of the division of a network into modules.

In addition, the network resilience to random failure and to targeted attack was evaluated. Network resilience is associated with the stability of a complex network to acute and focal network damage [14]. Random failure was assessed by randomly removing one node from the network and measuring changes repetitively, whereas targeted attack was assessed by removing the nodes in rank order of decreasing nodal BC. As for the statistical regional comparison between groups, the false discovery rate was estimated to assess the regional difference in BC, degree distribution, and clustering of connectivity between two groups for each comparison. All combinations of pre-CC, post-CC, and control groups were compared. Graph Analysis Toolbox (GAT) (https://www.nitrc.org/prijects/gat/) with SPM8 running on MATLAB 2014a was used for the graph theoretical analysis in this study. The analysis is detailed elsewhere [22].

### Statistical analysis

Comparisons were performed for the areas under the curve (AUCs) of each network measure and the resilience of the groups using GAT. To test the significance, the observed between-group difference in the AUC for each network measure was placed in the corresponding permutation distribution, and the p-value was calculated based on its percentile position. A one-tailed nonparametric permutation test (1000 repetitions) was performed for evaluation of the regional differences in BC and clustering between the two groups for each comparison. To correct for multiple comparisons, a false discovery rate p<0.05 was deemed significant.

For the clinical demographics, the differences in clinical parameters among the three groups were evaluated using one-way ANOVA for age at examination, Pearson’s χ^2^ test for sex and seizure frequency, and paired *t*-test for antiepileptic drugs and IQ/DQ outcome with JMP 10 (SAS Institute Inc., Cary, NC).

## Results

### Demographics

Mean ages of the pre-CC, post-CC, and control groups were 8.5 (SD 4.2), 9.0 (SD 4.4), and 9.2 (SD 4.3) years, respectively. Sex (male to female) ratios of the CC and control groups were 11:10 and 13:19, respectively. There were no significant differences in the mean age and sex among the three groups (p = 0.8401 and p = 0.6044, respectively). Participants’ postoperative outcomes are shown in Table 1. There was no significant difference in the mean number of antiepileptic drugs between the pre-CC and post-CC groups (p = 0.9423). There was a significant reduction in seizure frequency or the mean total numbers of seizures per month between the pre- and post-CC groups (p = 0.0019). There were four patients (19.0%) with seizure freedom, eight patients (38.1%) with a > 50% reduction in seizures, and nine patients (42.9%) with a < 50% reduction 1 year after CC. There was no relationship in the mean IQ/DQ levels between the pre- and post-CC groups 1 year after the CC (p = 0.8898).

**Table 1:** Participant’s postoperative outcomes.

Features	Preoperative data	Outcome
**Total number of seizures per month (mean ± SD)**	328.0 ± 235.6	150.4 ± 234.0
**Total number of antiepileptic drugs at examination (mean ± SD)**	3.2 ± 0.8	3.1 ± 0.7
**IQ/DQ outcome (mean ± SD)**	25.0 ± 20.8	24.5 ± 22.2
**One-year postoperative seizure outcome (no.)**		
Seizure free		4
>50% reduction		8
<50% reduction		9

DQ, developmental quotient; IQ, intelligence quotient; no., number; SD, standard deviation.

### Morphological analysis

Gray matter volume was not significantly changed between the pre-CC and post-CC groups and between the pre-CC and control groups.

### Graph analysis

**Fig 1:**
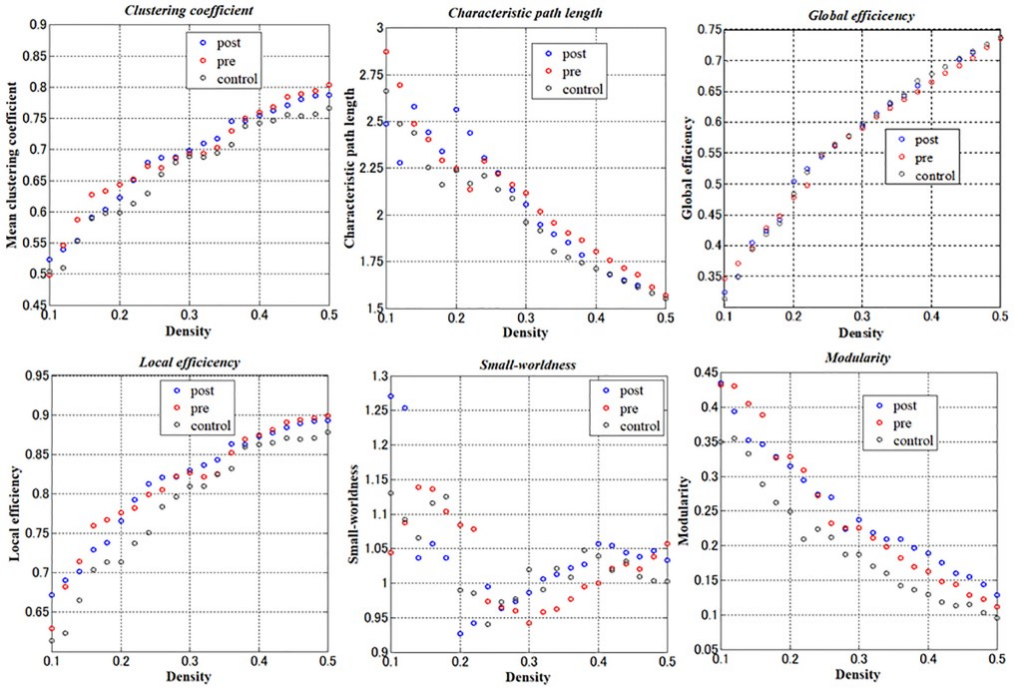
Results of AUC comparisons in each network metrics among the three groups. The meanings of the measures are described in the Materials and Methods section of the text. Each point is the mean value of global network metrics for the pre-CC, post-CC, and control groups across the range of density thresholds from 0.1 to 0.5 used to binarize the adjacency matrix. There was no relationship in global graph metrics among the three groups. AUC, area under the curve.

**Fig 2:**
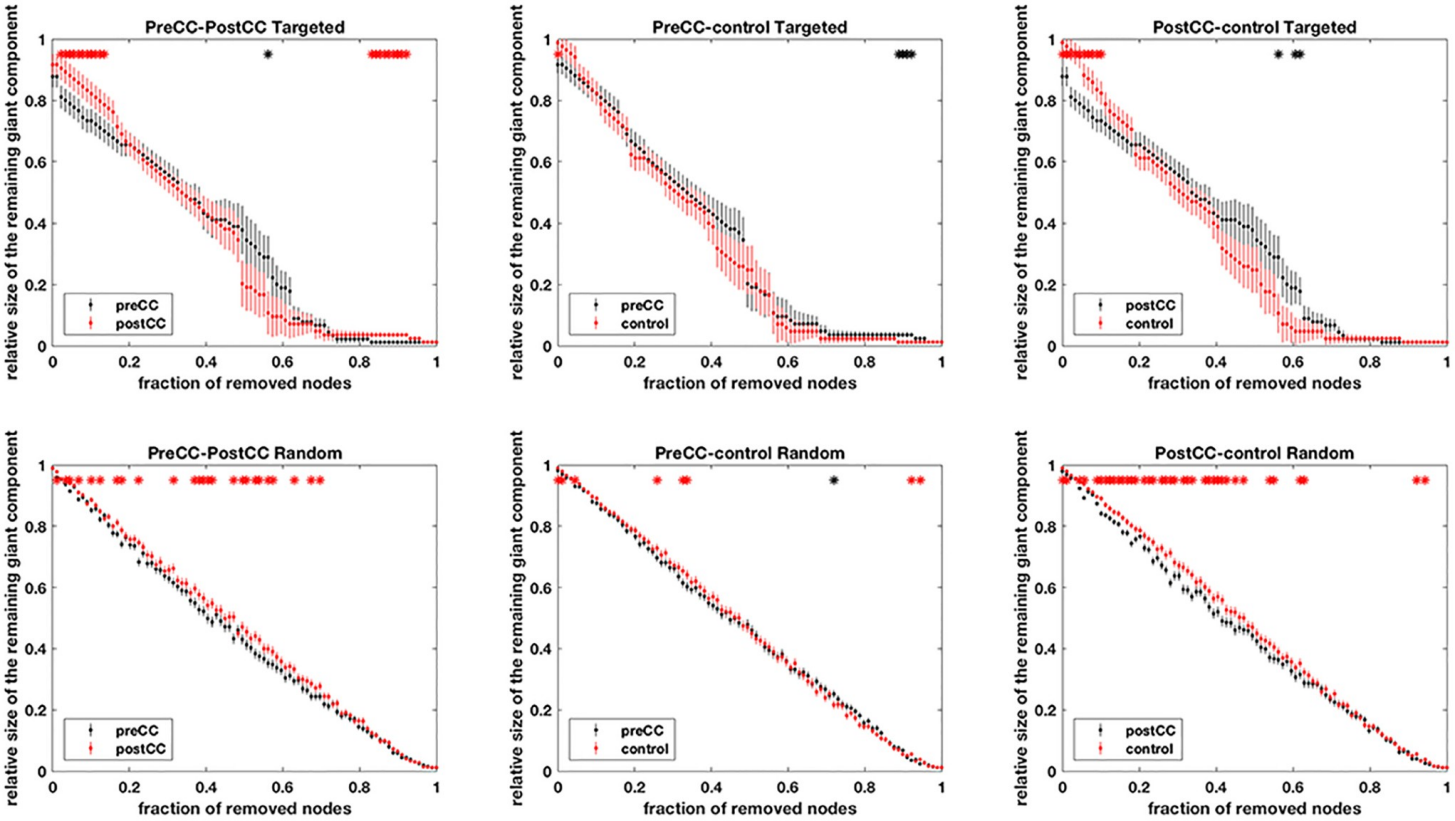
Results of assessments of network resilience to targeted attack or random failure among the three groups. The p-values of AUC comparisons between pre-CC and post-CC networks, between control and pre-CC networks, and between control and post-CC networks are shown in each targeted attack or random failure analysis. The trajectories of the relative size of the largest components as the principal nodes are demonstrated and each error bar represents the standard distribution for repetitive permutations. The asterisks denote significant differences between groups (p<0.05). The post-CC group showed significantly lower resilience to random failure than the pre-CC group and control group.

The results of network metrics are shown in Fig 1 and S2–S4 Figs. There was no relationship in clustering coefficient, small-worldness, and modularity among the three groups. The results of network resilience to random failure or targeted attack are presented in Fig 2. There was no relationship in the resilience to targeted attack among the three groups. However, the post-CC group showed significantly lower resilience to random failure than the pre-CC group and control group (p = 0.0450 and p = 0.0200, respectively).

**Fig 3:**
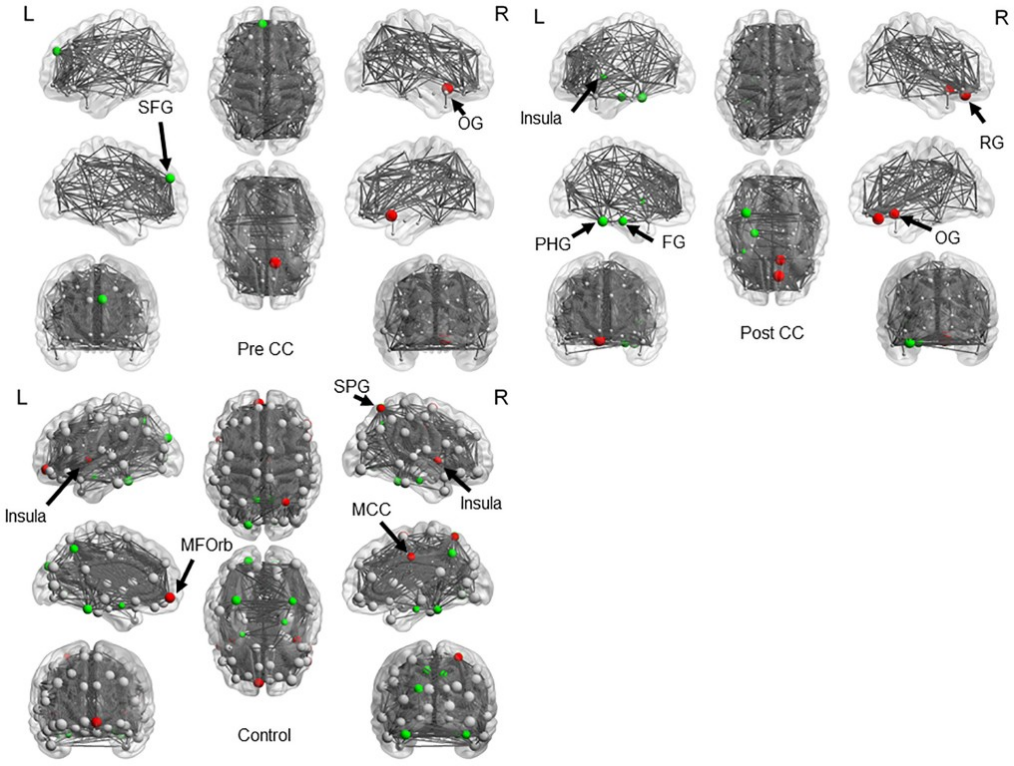
Results of network hub nodes and edges. Red denotes hubs with BC > 2 SD and green denotes those with BC > 1 SD. BC, betweenness centrality; SD, standard deviation; SFG, superior frontal gyrus; OG, olfactory gyrus; PHG, parahippocampal gyrus; FG, fusiform gyrus; RG, rectus gyrus; MFOrb, middle orbitofrontal gyrus; SPG, superior parietal gyrus; MCG, middle cingulate gyrus; L, left; R, right.

**Fig 4:**
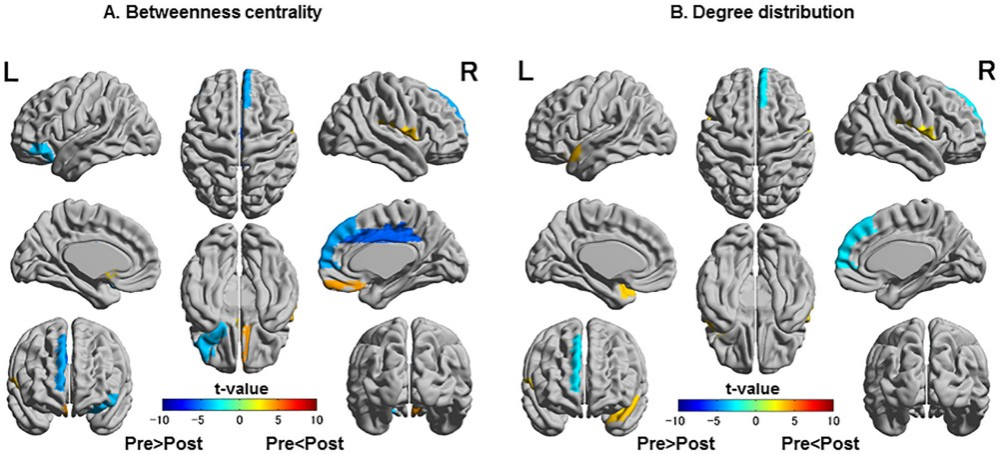
Regional comparison of betweenness centrality and degree distribution between pre-CC and post-CC groups. The pseudocolor scale represents t-values for betweenness centrality and degree distribution. Colored areas denote significant differences (false discovery rate p<0.05). CC, corpus callosotomy; L, left; R, right.

The network nodes, edge locations, and the size of BC in the three groups are presented in Fig 3 and Table 2. The locations of hub nodes > 2 SD were limited to the right olfactory gyrus in the pre-CC group and the right olfactory gyrus and rectus gyrus in the post-CC group. At a more liberal criterion of 1 SD, significant hub node locations expanded postoperatively to include the left insula, left fusiform gyrus, and left parahippocampal gyrus, although the left frontal gyrus that had been observed preoperatively was missed. Significant hub node locations in the control group were the bilateral insula, right middle cingulate and superior parietal gyri, and left middle frontal gyrus at 2 SD, and the right hippocampal and fusiform gyri, left amygdala, and left fusiform and superior occipital gyri additionally at 1 SD (Fig 3, Table 2). The regional comparison in BC revealed decreased connectivity in the right middle cingulate gyri and medial side of the right superior frontal gyrus in the post-CC group (Fig 4A). The regional comparison in degree distribution also revealed decreased connectivity in the medial side of the right superior frontal gyrus in the post-CC group (Fig 4B). There was no change in local connectivity in the thalamus and basal ganglia.

**Table 2:** Locations of network hub nodes with BC values greater than 1 or 2 SD in the three groups.

	Nodes > 2 SD	Nodes > 1 SD
**Pre-CC**		
Right	Olfactory	Olfactory
Left		Superior frontal
**Post-CC**		
Right	Olfactory	Olfactory
	Rectus	Rectus
Left		Fusiform
		Insula
		Parahippocampal
**Control**		
Right	Insula	Fusiform
	Middle cingulate	Insula
	Superior parietal	Middle cingulate
		Parahippocampal
		Superior parietal
Left	Insula	Amygdala
	Middle frontal	Fusiform
		Insula
		Middle frontal
		Superior occipital

BC, betweenness centrality; CC, corpus callosotomy; SD, standard deviation.

## Discussion

This is the first study, to our knowledge, to focus on alterations in the anatomical covariance network after CC using 3D sagittal T1-weighted magnetization-prepared MRI. We performed graph theoretical analysis to evaluate differences in the anatomical covariance network between pre- and post-CC patients as well as in each CC group and the control group. The results suggested that post-CC patients had more common hub node locations with control children compared with pre-CC patients but decreased degree distribution and BC in the medial side of the superior frontal gyrus, decreased BC in the middle cingulate gyri, and lower resilience to random failure.

No change in global network efficiency among the three groups was found in the present epilepsy patients. Previous studies taking graph theoretical approaches contrarily revealed an impaired anatomical covariance network affecting global networks in patients with generalized or focal epilepsy and stronger structural global connectivity and modularity with excessive network robustness versus controls [15, 19]. Fewer edges, weaker clustering coefficients, and longer path lengths in structural networks constructed using DTI are related to lower cognitive performance in adult patients with chronic epilepsy [24]. On resting-state functional MRI (fMRI), default mode and salience networks comprise weaker hubs in patients with medically intractable localization-related epilepsy compared with age- and sex-matched typically developing children [25]. While the present participants, as patients who had undergone palliative surgery, had similar backgrounds to those reported in a population-based study in Sweden [1], they had more severe intellectual disability and cognitive dysfunction and higher seizure frequency than epilepsy patients in previous studies. Differences in global network efficiency among the three groups resulted from methodology, epilepsy etiology, and frequency.

With respect to the effects of CC, the alteration in the BC distribution after CC was associated with the reappearance of normal hub nodes. Hub nodes in patients appeared after CC in the right rectus gyrus, and nodes marked by BC values 1 SD greater than the mean appeared in the left insula, fusiform gyrus, and parahippocampal gyrus. The BC-defined node locations in the patient group comprised a subset of the BC-defined nodes with values more than 1 SD in the control group (see Table 2). We interpret the results in terms of the appearance of a normal network structure in the patient group after CC. We also postulate an assumption in line with the functional network change following surgically resective control of seizures in an LGS patient [26]. In the postoperative functional connectivity of a boy with LGS, there were greater between-network negative connectivity and stronger within-network positive connectivity, a pattern that more closely resembles the network behavior typically seen in healthy children and adults. Regional network abnormalities were estimated mainly in the superior frontal gyrus before CC. Corpus callosum resection induced an improvement in pathological connectivity.

The other effect of CC was a division of pathological paths. The values of both BC and degree distribution decreased postoperatively in the median line, although there was a discrepancy between the two metrics: while the BC was mainly reduced in the middle cingulate gyrus, the degree distribution was mainly reduced in the superior frontal gyrus. The lower median line after CC is consistent with the functional network results in patients with LGS in a 19-channel electroencephalography study [8, 9], in which the hub moved from the paramedian regions to both hemispheres, including lateral regions, and their seizures decreased. Resting-state fMRI in patients with medically refractory epilepsy also revealed a marked reduction in a large part of interhemispheric functional connectivity after CC, despite residual connectivity of primary sensorimotor and visual areas [27]. In other words, a lowered median connectivity after CC was confirmed in both structural and functional network measures. A decreased clustering coefficient in the delta band has been reported in functional global network measures [8, 9]. While the reported global connectivity alteration may not appear to match the present results, the apparent discrepancy in the global network measures may stem from functional reorganization and structural change, a better seizure outcome after CC in the previous reports [8, 9], or a higher number of ROIs in the present study.

The altered structural and functional connectivity after surgery correlated with seizure frequency in the aforementioned articles. In our study, alteration of the anatomical covariance network after CC was associated with subsequent seizure reduction. In this vein, while the bilateral olfactory cortex remained the hub after CC, a hub in the bilateral rectus gyrus appeared postoperatively in place of the preoperative left superior frontal gyrus. Among the normal hubs observed in the control group, the left insula and left fusiform gyri appeared in the post-CC group. There may also be a relationship between the left parahippocampal hub in the post-CC group and the right parahippocampal hub in the control group. In addition, the interhemispheric connections to and from the right middle cingulate gyrus represented by BC seem to have been sloughed after dissection of the corpus callosum, although a hub was found there in the control group. This may mean that selective resection of the corpus callosum not only dissected the pathological path-propagated epileptic discharges, but also recovered the non-pathological hub node.

There was no postoperative change in the gray matter volume of the thalamus and in the anatomical covariance between the thalamus and the cortex. A significant role of thalamocortical pathways was previously demonstrated in patients with LGS and other generalized epilepsies [28, 29, 30]. Studies using EEG-fMRI revealed thalamic activation associated with epileptic discharges in patients with primary (idiopathic generalized syndromes) and secondary (focal epilepsy) bilateral synchrony [28, 29, 30]. Slow spike-and-wave discharges in LGS patients showed a mixture of increased and decreased resting-state fMRI activity, with an increase in the association cortex and thalamus followed by a prominent post-event reduction [31]. We speculate that the differences in study results arose from differences in methodology [11].

Postoperative network-based anatomical covariance showed significantly lower resilience to random failure than the preoperative network and that of children without a central nervous system abnormality. The human brain network based on fMRI is characterized by having approximately the same resilience to random failure as random and scale-free networks and considerably greater resilience to targeted attack than the scale-free network [32]. In another fMRI study with healthy participants, stronger brain resilience to targeted and random attacks correlated with a higher individual IQ, and the relevant regions with resilience were language and memory network areas [33]. According to previous studies, resilience analyses including anatomical covariance revealed significantly lower resilience in the gray matter networks of adult idiopathic generalized epilepsy patients to both random failure and targeted attack compared with healthy adults [16]. Pediatric patients with intractable epilepsy also showed an association between the resilience of the network topologies of fMRI to interictal discharges and stronger resting-state network connectivity and network vulnerability to interictal discharges and worse neurocognitive outcomes [34]. While severing of the corpus callosum induced not only a postoperative alteration in the degree distribution, but also a postoperative reduction in anatomical covariance, it additionally caused postoperative network vulnerability irrespective of the unchangeable IQ.

There are, however, several limitations to this study. First, the number of patients analyzed in the study was small. Second, the effect of CC was evaluated specifically in patients without a detectable lesion on MRI. There may be a difference in structural networks based on anatomical covariance in patients with a detectable MRI lesion. Third, all patients with epilepsy had been taking several kinds of antiepileptic drugs, which could have affected the results by altering brain function to various degrees. Patients prescribed carbamazepine and oxcarbazepine have lower BC in brain networks on graph theoretical analysis [35]. Fourth, the clinical characteristics of patients with epilepsy were insufficient in two areas: postoperative IQ/DQ evaluation was conducted too early, and maladaptive behavioral data were unavailable. Fifth, the IQ or DQ of the control group of school-aged patients was not evaluated, although the control children were confirmed neither to be receiving nor to have received special support education. Sixth, we could not preclude the possibility that a small injury to the cingulate gyrus, caused by instrument retraction during the surgical approach, induced a functional change, with a significant effect in a small number of patients biasing the mean. Checked by two experts, there was no structural abnormality on postoperative MRI, including 3D sagittal T1-weighted images, transverse conventional T1-weighted images, transverse turbo spin echo T2-weighted images, and coronal fluid-attenuated inversion recovery images. In addition, neurosurgeons ensured that the arachnoid membrane of the interhemispheric fissure was sharply dissected, in line with the standard method [36], and that there was no visible injury or bleeding in the cingulate gyrus in all cases. This was a methodological problem, although we attempted to remove bias. Finally, binary thresholding is a common method but could be a cause of bias. Inclusion of low thresholds is likely to include effects from false positives while inclusion of very high thresholds will include measurements of highly disconnected networks that are not reflective of the true connectome. Briefly, there can be a failure to detect true group effects if the effect is only manifested in a limited range of thresholds [37]. We selected the range of 0.10 to 0.50 at intervals of 0.02 as thresholds, which were the same as in previous articles. In particular, Hosseini et al. [22], who made the GAT package, investigated their between-group differences on networks thresholded at a range of densities from 0.15 to 0.45.

Despite these methodological limitations, the present study provides novel observations on epilepsy alterations in children after CC.

## Conclusions

Graph theoretical analysis of anatomical covariance derived from structural imaging revealed two structural network effects of CC related to a lower seizure frequency. One was a partial shift in the BC distribution to the normal location and the other was a reduced BC and degree distribution along the median line including the middle cingulate gyrus. The structural network showed significantly lower resilience to random failure after CC than before CC and compared with controls. Voxel-based morphometry did not reveal a significant effect of CC.

## Supporting information

S1 Fig Corpus callosotomy in pediatric patients with intractable epilepsy treated at the National Center for Neurology and Psychiatry from 2010 to 2017. (TIF)

S2 Fig Results of between-group differences between the pre-CC and post-CC networks and the 95% confidence intervals in global network measures. The * marker (red) shows the difference between the pre-CC and post-CC networks. Any * markers (blue) overshooting the confidence intervals indicate densities at which the difference is significant. No trials were statistically significant.
(TIF)

S3 Fig Results of between-group differences between control and the pre-CC networks and the 95% confidence intervals in global network measures. The * marker (red) shows the difference between control and the pre-CC networks. Any * markers (blue) overshooting the confidence intervals indicate densities at which the difference is significant. No trials were statistically significant.
(TIF)

S4 Fig Results of between-group differences between control and the post-CC networks and the 95% confidence intervals in global network measures. The * marker (red) shows the difference between control and the post-CC networks. Any * markers (blue) overshooting the confidence intervals indicate densities at which the difference is significant. No trials were statistically significant.
(TIF)

S1 Appendix The input data of the pre-CC group to GAT. (ZIP)

S2 Appendix The input data of the post-CC group to GAT. (ZIP)

S3 Appendix The input data of the control group to GAT. (ZIP)

S4 Appendix Demographics of three groups. (ZIP)

S5 Appendix The results of graph theoretical analysis of preCC and postCC analysis. (ZIP)

S6 Appendix The results of graph theoretical analysis of control and preCC analysis. (ZIP)

S7 Appendix The results of graph theoretical analysis of control and post CC analysis. (ZIP)

S8 Appendix VBM_preCC vs control analysis. The input data for the comparison between the pre-CC and control groups to CAT12.
(ZIP)

S9 Appendix VBM_preCC vs postCC analysis. The input data for the longitudinal comparison between the pre- and post-CC groups to CAT12.
(ZIP)
