# Supplementary figures and images for: Alteration of the anatomical covariance network after corpus callosotomy in pediatric intractable epilepsy
Source: PLoS One. 2019 Dec 5;14(12):e0222876. doi: 10.1371/journal.pone.0222876 (PMC6894802; doi:10.1371/journal.pone.0222876)

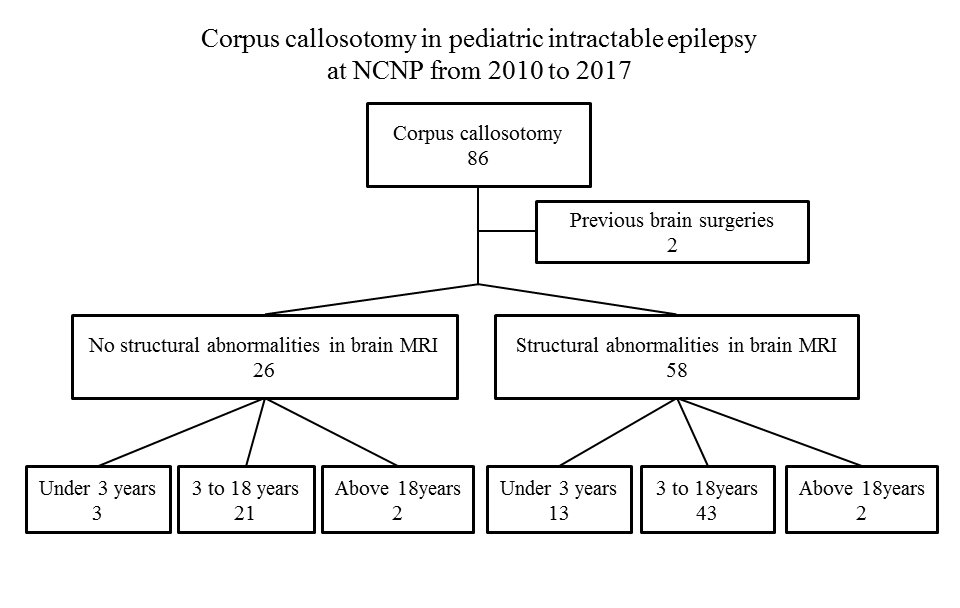

Supplement: S1 Fig — (TIF) [file pone.0222876.s001.tif]

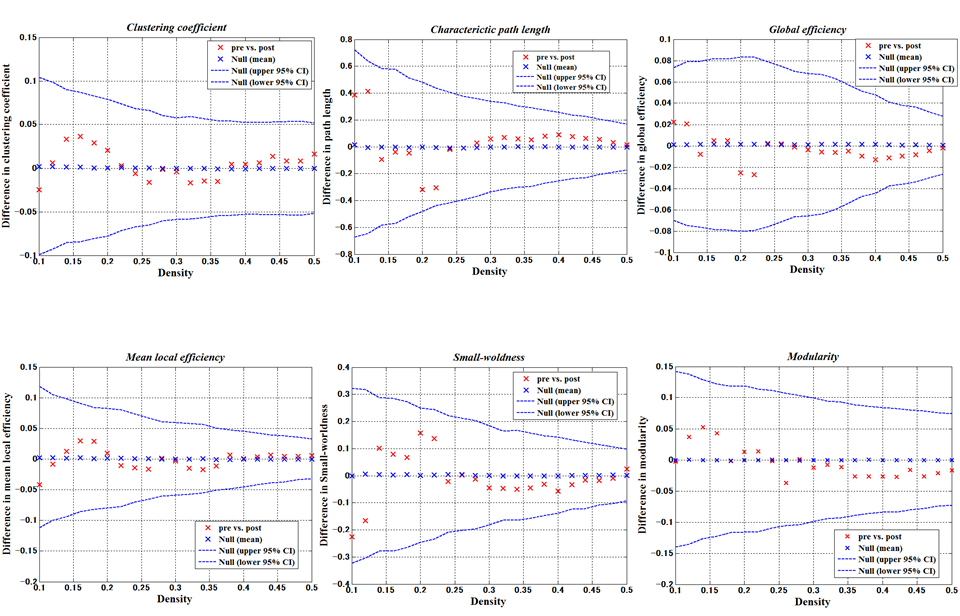

Supplement: S2 Fig — The * marker (red) shows the difference between the pre-CC and post-CC networks. Any * markers (blue) overshooting the confidence intervals indicate densities at which the difference is significant. No trials were statistically significant. (TIF) [file pone.0222876.s002.tif]

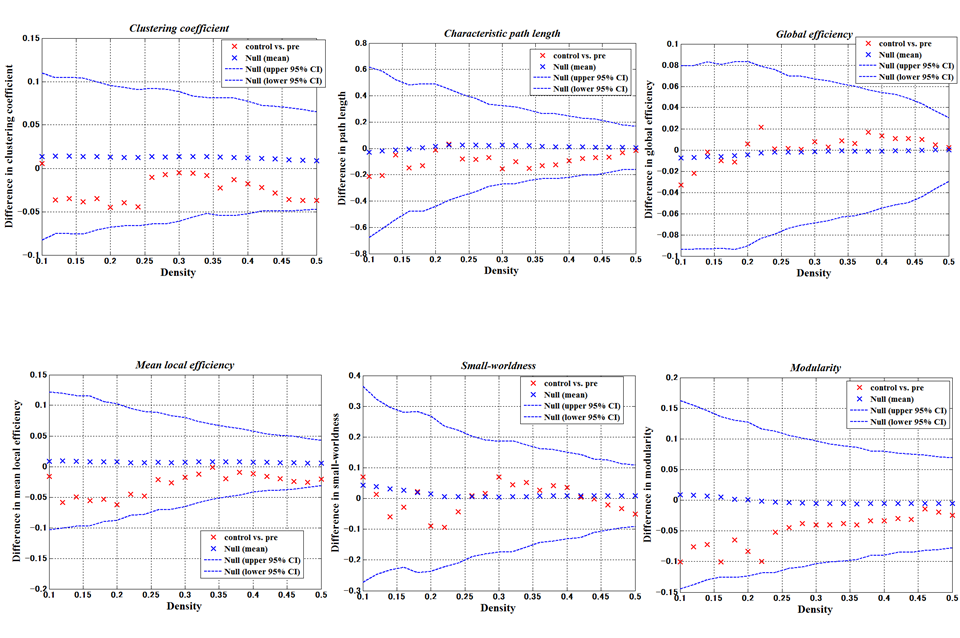

Supplement: S3 Fig — The * marker (red) shows the difference between control and the pre-CC networks. Any * markers (blue) overshooting the confidence intervals indicate densities at which the difference is significant. No trials were statistically significant. (TIF) [file pone.0222876.s003.tif]

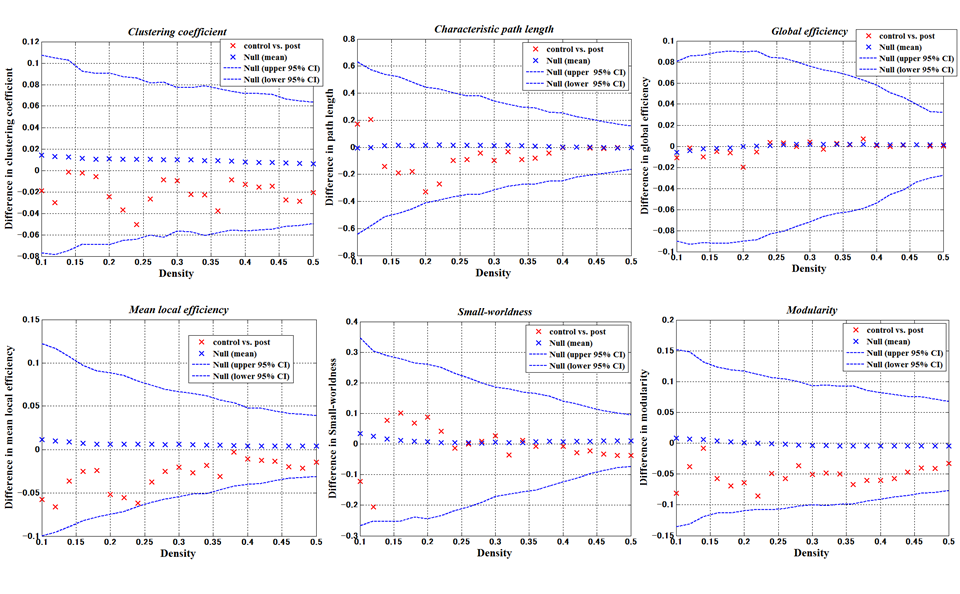

Supplement: S4 Fig — The * marker (red) shows the difference between control and the post-CC networks. Any * markers (blue) overshooting the confidence intervals indicate densities at which the difference is significant. No trials were statistically significant. (TIF) [file pone.0222876.s004.tif]
